# Supplementary material for: Farmer vs. Industrial Practices: Impact of Variety, Cropping System and Process on the Quality of Durum Wheat Grains and Final Products
Source: Foods. 2023 Mar 3;12(5):1093. doi: 10.3390/foods12051093 (PMC10000652; doi:10.3390/foods12051093)
Supplement: Supplementary file 1 [file foods-12-01093-s001.zip › foods-2196363-supplementary.pdf]

Supplementary data

Table S1. Protein composition measured on lab-milled grain using SE-HPLC analysis: protein fractions from F1 to F5 (as a percentage of the total protein content), Fi (SDS-unextractable polymeric proteins as a percentage of the total protein content), UPP% (100 × Fi/(F1 + F2 + Fi)) and the gliadin/glutenin ratio (F4/F1 + F2 + Fi) and total protein content (% db). Values are the mean of replicates.

Variety	Group	Cropping System	Location	Year of harvest	SE-HPLC protein fraction (% of total protein content)						UPP (%)	Glia/Glu	Total protein content (% db)
					F1	F2	F3	F4	F5	Fi			
Anvergur	IND	ZI	Mauguio	2016	7.0 a	22.6 bcde*	13.1 a	28.3 cdef	22.5 a	6.4 cde	17.8 bc	0.78 g	7.4 de
Bidi17	FAR	ZI	Mauguio	2016	7.4 a	23.3 abcd	11.9 a	29.6 abc	22.5 a	5.3 defg	14.7 cde	0.82 e	8.8 bc
Bidi17_2	FAR	ZI	Mauguio	2016	7.9 a	23.6 abc	11.3 a	30.7 ab	22.7 a	3.7 hi	10.6 f	0.87 c	9.3 ab
Claudio	IND	ZI	Mauguio	2016	6.2 a	20.6 fg	12.3 a	29.8 abc	22.7 a	8.5 a	24.0 a	0.84 d	7.3 e
Clovis	DIV	ZI	Mauguio	2016	7.9 a	22.6 bcde	11.9 a	27.6 ef	23.3 a	6.7 bcd	18.0 bc	0.74 h	7.5 de
Desire	DIV	ZI	Mauguio	2016	7.5 a	21.7 def	12.5 a	29.7 abc	22.7 a	5.8 cdef	16.5 c	0.85 d	7.4 de
Fabulis	IND	ZI	Mauguio	2016	7.4 a	24.0 abc	12.3 a	28.5 cde	22.3 a	5.4 defg	14.8 cde	0.77 g	8.8 bc
Joyau	IND	ZI	Mauguio	2016	8.0 a	22.4 cde	12.1 a	28.0 def	21.5 a	7.9 ab	20.6 b	0.73 i	7.6 de
Karur	IND	ZI	Mauguio	2016	7.1 a	23.5 abcd	12.1 a	28.7 cde	21.9 a	6.6 cd	17.8 bc	0.77 g	7.3 de
LA1823	FAR	ZI	Mauguio	2016	7.2 a	24.2 ab	12.5 a	30.3 ab	21.4 a	4.3 fghi	12.0 def	0.85 d	8.9 abc
Meliani1B	DIV	ZI	Mauguio	2016	6.4 a	21.3 efg	12.9 a	30.3 ab	23.6 a	5.4 defg	16.4 c	0.91 b	7.8 de
Miradoux	IND	ZI	Mauguio	2016	6.6 a	23.2 abcd	14.0 a	28.0 def	23.2 a	4.9 efgh	14.2 cde	0.81 f	7.2 e
Oued Zenati	FAR	ZI	Mauguio	2016	6.8 a	23.3 abcd	12.1 a	31.2 a	23.2 a	3.3 i	9.9 f	0.93 a	9.1 abc
Pescadou	IND	ZI	Mauguio	2016	7.4 a	24.5 a	12.1 a	28.3 cdef	22.0 a	5.7 cdef	15.3 cd	0.75 h	8.2 cd
Qualidou	IND	ZI	Mauguio	2016	7.4 a	24.1 abc	12.9 a	29.5 bcd	22.0 a	4.1 ghi	11.4 ef	0.83 de	7.5 de
RG1110	DIV	ZI	Mauguio	2016	8.6 a	22.3 cde	11.6 a	30.6 ab	22.6 a	4.3 fghi	12.1 def	0.87 c	8.7 bc
RG218	DIV	ZI	Mauguio	2016	6.9 a	20.1 g	13.2 a	30.5 ab	22.2 a	7.0 bc	20.7 b	0.90 b	9.7 a
RG69649	DIV	ZI	Mauguio	2016	7.4 a	22.7 bcde	12.4 a	31.0 ab	23.1 a	3.4 i	10.4 f	0.93 b	9.7 a
Vivit	DIV	ZI	Mauguio	2016	7.3 a	22.4 cde	13.0 a	27.0 f	24.0 a	6.4 cde	17.7 bc	0.75 h	7.4 de
mean	DIV	ZI	Mauguio	2016	7.4 a	22.0 c	12.5 a	29.5 b	23.1 a	5.6 b	16.0 a	0.85 a	8.3 b
mean	FAR	ZI	Mauguio	2016	7.3 a	23.6 a	12.0 a	30.5 a	22.4 ab	4.2 c	11.8 b	0.87 a	9.0 a
mean	IND	ZI	Mauguio	2016	7.1 a	23.1 b	12.6 a	28.6 c	22.3 b	6.2 a	17.0 a	0.79 b	7.7 c
Mean ± SD	*all*	*ZI*	*Mauguio*	*2016*	*7.3 ± 0.58*	*22.8 ± 1.19*	*12.4 ± 0.64*	*29.4 ± 1.27*	*22.6 ± 0.69*	*5.5 ± 1.47*	*15.5 ± 3.90*	*0.8 ± 0.06*	*8.2±0.89*
CV%					*8.0%*	*5.2%*	*5.1%*	*4.3%*	*3.0%*	*26.5%*	*25.1%*	*7.8%*	*10.8%*
Anvergur	IND	all	Mauguio	2017	7.0 bcde	18.4 fgh	9.3 b	35.7 defghi	17.7 cd	11.8 a	31.8 bc	0.96 def	10.0 gh
Bidi17	FAR	all	Mauguio	2017	7.0 bcde	18.9 defg	9.3 b	37.9 c	17.2 d	9.7 cd	27.1 fgh	1.06 c	11.9 c
Bidi17_2	FAR	all	Mauguio	2017	7.9 ab	19.5 bc	9.1 b	36.4 cdefg	18.2 bcd	8.9 d	24.5 h	1.00 cdef	11.7 c
Claudio	IND	all	Mauguio	2017	5.8 e	16.3 j	8.6 b	39.2 b	18.1 bcd	11.8 a	34.6 a	1.16 b	10.0 gh
Clovis	DIV	all	Mauguio	2017	7.4 abcd	19.7 ab	9.2 b	34.0 i	18.0 bcd	11.6 ab	30.0 cde	0.88 gh	9.7 h
Desire	DIV	all	Mauguio	2017	6.8 bcde	18.3 gh	9.1 b	37.0 cde	18.4 bcd	10.4 bc	29.1 defg	1.04 cd	10.4 ef
Fabulis	IND	all	Mauguio	2017	6.5 bcde	19.4 bcd	9.5 b	34.3 hi	19.9 a	10.4 bc	28.8 defg	0.95 efg	9.7 h
Joyau	IND	all	Mauguio	2017	6.5 bcde	19.0 cdef	9.6 b	35.2 efghi	17.8 cd	11.9 a	31.9 bc	0.94 efg	10.4 ef
Karur	IND	all	Mauguio	2017	6.1 de	18.8 efg	8.3 b	35.6 efghi	19.0 abc	12.1 a	32.9 ab	0.96 def	10.2 fg
LA1823	FAR	all	Mauguio	2017	7.7 abc	20.1 a	9.2 b	36.8 cdef	17.1 d	9.0 d	24.6 h	1.00 cdef	10.8 e
Meliani 1B	DIV	all	Mauguio	2017	6.2 cde	17.4 i	9.2 b	40.6 a	17.3 d	9.3 cd	28.2 defg	1.24 a	11.3 b
Miradoux	IND	all	Mauguio	2017	7.9 ab	18.9 defg	9.1 b	35.4 efghi	19.1 abc	9.7 cd	26.6 fgh	0.97 def	12.3 b
OuedZenati	FAR	all	Mauguio	2017	6.7 bcde	19.7 ab	9.3 b	37.6 cd	17.3 d	9.5 cd	26.4 gh	1.05 cd	10.3 fg
Pescadou	IND	all	Mauguio	2017	8.6 a	20.2 a	8.8 b	33.9 i	17.3 d	11.2 ab	27.7 efg	0.85 h	11.2 d
Qualidou	IND	all	Mauguio	2017	8.7 a	19.8 ab	8.4 b	36.1 cdefg	19.5 ab	7.4 e	20.6 i	1.01 cdef	10.3 fg
RG1110	DIV	all	Mauguio	2017	6.7 bcde	18.0 h	10.2 b	37.5 cd	17.2 cd	10.3 bc	29.5 cdef	1.07 c	13.0 a
RG218	DIV	all	Mauguio	2017	6.5 bcde	18.5 fg	12.7 a	35.0 fghi	18.1 bcd	9.2 cd	27.0 fgh	1.02 cde	13.1 a
RG69649	DIV	all	Mauguio	2017	7.7 abc	19.3 bcde	8.2 b	36.7 cdefg	17.2 d	10.9 ab	28.8 defg	0.97 def	12.5 b

Vivit	DIV	all	Mauguio	2017	7.2 bcde	18.8 efg	9.4 b	34.8 ghi	18.3 bcd	11.5 ab	30.6 bcd	0.93 fg	10.8 e
mean	DIV	all	Mauguio	2017	6.9 a	18.6 c	9.7 a	36.5 b	17.8 b	10.5 a	29.0 a	1.02 a	11.6 a
mean	FAR	all	Mauguio	2017	7.3 a	19.6 a	9.2 b	37.2 a	17.4 b	9.3 b	25.b b	1.03 a	11.7 a
mean	IND	all	Mauguio	2017	7.1 a	18.8 b	9.0 b	35.7 c	18.6 a	10.8 a	29.3 a	0.98 b	10.2 b
all	all	ZI	Mauguio	2017	6.9 b	18.5 b	9.2 a	36.1 b	18.3 a	11.1 a	30.3 a	0.99 b	10.8 b
all	all	LI	Mauguio	2017	7.3 a	19.3 a	9.4 a	36.6 a	17.8 b	9.6 b	26.6 b	1.01 a	11.3 a
Mean ± SD	*all*	*all*	*Mauguio*	*2017*	*7.1 ± 0.87*	*18.9 ± 1.11*	*9.3 ± 0.99*	*36.3 ± 1.90*	*18.1 ± 0.95*	*10.3 ± 1.72*	*28.5 ± 4.27*	*1.0 ± 0.10*	*11.0±1.19*
CV%					*12.3%*	*5.9%*	*10.7%*	*5.2%*	*5.3%*	*16.6%*	*15.0%*	*9.7%*	*10.8%*
Anvergur	IND	ZI	all	2019	7.0 b	20.4 b	10.3 a	31.6 b	20.7 a	10.0 b	26.7 b	0.85 c	9.1 b
Bidi17	FAR	ZI	all	2019	7.4 ab	20.9 b	10.3 a	33.8 a	20.2 a	7.4 c	20.5 c	0.95 a	10.6 a
Claudio	IND	ZI	all	2019	6.0 c	18.0 c	9.8 b	33.5 a	20.5 a	12.1 a	33.6 a	0.93 ab	9.4 b
LA1823	FAR	ZI	all	2019	7.7 a	21.9 a	10.3 a	33.3 a	19.6 a	7.1 c	19.3 c	0.91 b	9.9 b
mean	FAR	ZI	all	2019	7.6 a	21.3 a	10.3 a	33.6 a	19.9 a	7.2 b	20.0 b	0.93 a	10.2 a
mean	IND	ZI	all	2019	6.5 b	19.2 b	10.0 b	32.6 b	20.6 a	11.1 a	30.2 a	0.89 b	9.3 b
all	all	ZI	Mauguio	2019	6.8 b	20.0 a	10.0 b	33.7 a	19.8 b	9.7 a	26.4 a	0.92 a	10.8 a
all	all	ZI	Purpan	2019	7.3 a	20.6 a	10.3 a	32.6 b	20.7 a	8.5 a	23.3 a	0.90 b	8.8 b
Mean ± SD	*all*	*ZI*	*all*	*2019*	*7.0 ± 0.74*	*20.3 ± 1.60*	*10.2 ± 0.29*	*33.1 ± 1.26*	*20.2 ± 0.90*	*9.2 ± 2.35*	*25.3 ± 6.44*	*0.9 ± 0.05*	*9.8±1.35*

*Values followed by the same letter within each column are not significantly different based on Student-Newman-Keuls (SNK) test performed at $\alpha = 0.05$.

Table S2. Protein composition measured on stone-milled grain (semolina) using SE-HPLC analysis: protein fractions from F1 to F5 (as a percentage of the total protein content), Fi (SDS-unextractable polymeric proteins as a percentage of the total protein content), UPP% (100 × Fi/(F1 + F2 + Fi)) and the gliadin/glutenin ratio (Glia/Glu=(F4/(F1 + F2 + Fi)). Total protein content measured by Kjeldahl method (% db). Values are the mean of replicates.

Cultivar	Group	Cropping System	Year of harvest	SE-HPLC protein fraction (% of total protein content)						UPP (%)	Glia/Glu	Protein content (% db) (Kjeldhal)
				F1	F2	F3	F4	F5	Fi			
Anvergur	IND	all	2017	7.7 b*	19.7 a	9.1 a	36.0 b	17.0 a	10.4 b	27.5 b	1.0 b	11.3 d
Bidi17	FAR	all	2017	8.0 b	19.1 a	8.5 b	38.2 a	16.0 c	10.1 b	27.2 b	1.0 b	14.9 a
Claudio	IND	all	2017	6.0 c	16.8 b	8.5 b	39.2 a	16.7 b	12.9 a	36.1 a	1.1 a	12.1 c
LA1823	FAR	all	2017	8.3 a	20.3 a	8.4 b	36.7 b	16.7 b	9.7 b	25.2 b	1.0 b	12.6 b
mean	FAR	all	2017	8.2 a	19.7 a	8.5 b	37.5 a	16.4 b	9.9 b	26.2 b	0.99 a	13.7 a
mean	IND	all	2017	6.9 b	18.3 b	8.8 a	37.6 a	16.8 a	11.7 a	31.8 a	1.03 a	11.7 b
all	all	ZI	2017	7.6 a	18.9 a	8.6 a	37.0 a	17.1 a	10.8 a	29.1 a	1.00 a	12.5 b
all	all	LI	2017	7.5 a	19.1 a	8.7 a	38.0 a	16.1 b	10.7 a	28.9 a	1.02 a	12.9 a
all	all	all	2017	7.5 ± 0.96	19.0 ± 1.48	8.6 ± 0.44	37.5 ± 1.83	16.6 ± 0.80	10.8 ± 1.57	29.0 ± 4.76	1.01 ± 0.08	12.7 ± 1.48

*Values followed by the same letter within each column are not significantly different based on Student-Newman-Keuls (SNK) test performed at α = 0.05. FAR= varieties used by the peasant pasta makers, IND= varieties recommended by the semolina and pasta industry; ZI= zero input, LI=low input.

Table S3. Protein composition measured on dry pasta using SE-HPLC analysis: protein fractions from F1 to F5 (as a percentage of the total protein content), DTE-soluble (DTE soluble proteins as a percentage of the total protein content) and unextractable (unextractable proteins remaining after two extraction steps as a percentage of the total protein content).

Variety	Group	Cropping system	Process	Year of harvest	SE-HPLC fractions (% of total protein content)						
					F1	F2	F3	F4	F5	DTE-Soluble	Unextractable
Anvergur	IND	All	All	2017	1.6 a*	11.3 ab	8.6 a	26.6 b	12.9 a	37.8 a	1.2 b
Bidi17	FAR	All	All	2017	1.7 a	10.9 ab	8.2 a	26.7 b	12.8 a	34.2 b	5.5 a
Claudio	IND	All	All	2017	1.6 a	10.6 b	8.4 a	28.3 a	13.3 a	36.2 ab	1.7 b
LA1823	FAR	All	All	2017	1.8 a	11.8 a	8.0 a	27.3 a	13.7 a	34.9 b	2.5 b
Mean	FAR	All	All	2017	1.7 a	11.4 a	8.1 b	27.0 a	13.2 a	34.6 a	4.0 a
Mean	IND	All	All	2017	1.6 b	10.9 a	8.5 a	27.4 a	13.1 a	37.0 b	1.5 b
All	All	ZI	All	2017	1.7 a	10.8 b	8.0 b	26.6 b	13.5 a	35.9 a	3.5 a
All	All	LI	All	2017	1.7 a	11.5 a	8.6 a	27.8 a	12.8 b	35.7 a	1.9 b
All	All	All	Artisanal_2	2017	2.7 a	18.0 a	10.3 a	33.5 a	15.6 a	19.3 b	0.6 b
All	All	All	Industrial	2017	0.7 b	4.3 b	6.3 b	20.9 b	10.7 b	52.2 a	4.9 a
All	All	All	All	2017	1.8 a	11.9 a	8.8 a	28.0 a	13.3 a	33.7 a	2.4 a

* Values followed by the same letter within each column are not significantly different based on Student-Newman-Keuls (SNK) test performed at α = 0.05. FAR= varieties used by the peasant pasta makers, IND=varieties recommended by the semolina and pasta industry; ZI= zero input, LI=low input.

Table S4. Total protein content (% db) and protein composition measured on OCT cooked pasta using SE-HPLC analysis: SDS-soluble (SDS-soluble protein as a percentage of the total protein content), DTE-soluble (DTE soluble proteins as a percentage of the total protein content). Values are mean of replicates.

Variety	Group	Cropping System	Process	Year of harvest	Protein content (% db)	Protein fraction (% total protein content)	
						SDS-soluble	DTE-soluble
Anvergur	IND	ZI	Artisanal_1	2016	8.5 d	37.9* d	62.1 b
Bidi17	FAR	ZI	Artisanal_1	2016	9.0 b	43.8 b	56.2 d
Claudio	IND	ZI	Artisanal_1	2016	7.4 g	42.1 bc	57.9 cd
Fabulis	IND	ZI	Artisanal_1	2016	8.6 c	42.1 bc	57.9 cd
Joyau	IND	ZI	Artisanal_1	2016	8.3 e	35.1 e	64.9 a
Karur	IND	ZI	Artisanal_1	2016	7.2 h	34.3 e	65.7 a
LA1823	FAR	ZI	Artisanal_1	2016	9.1 a	34.5 e	65.5 a
Miradoux	IND	ZI	Artisanal_1	2016	7.2 h	44.7 b	55.3 d
Pescadou	IND	ZI	Artisanal_1	2016	7.9 f	47.9 a	52.1 e
Qualidou	IND	ZI	Artisanal_1	2016	7.9 f	40.6 c	59.4 c
mean	FAR	ZI	Artisanal_1	2016	9.1 a	39.2 b	60.9 a
mean	IND	ZI	Artisanal_1	2016	7.9 b	40.6 a	59.4 b
all	all	ZI	Artisanal_1	2016	8.1±0.69	40.3 ± 4.65	59.7 ± 4.65

* Values followed by the same letter within each column are not significantly different based on Student-Newman-Keuls (SNK) test performed at $\alpha = 0.05$.

Table S5. Total protein content measured by Kjeldahl method (% db). Protein composition measured on OCT cooked pasta using SE-HPLC analysis: SDS-soluble (SDS-soluble protein as a percentage of the total protein content), DTE-soluble (DTE soluble proteins as a percentage of the total protein content) and unextractable (unextractable proteins remaining after two extraction steps as a percentage of the total protein content). In vitro digested protein (% of the total protein content). Not determined: nd. Values are mean of duplicates.

Variety	Group	Year of harvest	Cropping system	Process	Protein content (% db)	Protein fraction (% total protein content)			In vitro digested proteins (%)
						SDS-soluble (%)	DTE-soluble (%)	Unextr. (%)	
Anvergur	IND	2016	ZI	Artisanal_1	8.5	37.9	62.1	0	nd
Bidi17	FAR	2016	ZI	Artisanal_1	9.0	43.8	56.2	0	nd
Claudio	IND	2016	ZI	Artisanal_1	7.4	42.1	57.9	0	nd
LA1823	FAR	2016	ZI	Artisanal_1	9.1	34.5	65.5	0	nd
Anvergur	IND	2017	All	Artisanal_2	12.5	32.9	60.3	6.8	31.5
Anvergur	IND	2017	All	Industrial	11.9	20.2	71.0	8.9	27.5
Bidi17	FAR	2017	All	Artisanal_2	15.0	31.2	60.5	8.4	38.0
Bidi17	FAR	2017	All	Industrial	14.4	20.2	67.3	12.5	30.5
Claudio	IND	2017	All	Artisanal_2	14.0	27.2	54.6	18.3	39.5
Claudio	IND	2017	All	Industrial	12.2	20.1	68.5	11.4	26.5
LA1823	FAR	2017	All	Artisanal_2	13.0	29.5	65.5	5.0	35.5
LA1824	FAR	2017	All	Industrial	12.6	19.3	71.9	8.7	29.7
All	All	2017	ZI	All	12.9 a*	24.5 a	64.4 a	11.1 a	32.1 a
All	All	2017	LI	All	13.4 a	25.6 a	65.5 a	8.9 a	32.6 a
All	All	2017	All	Artisanal_2	13.7 a	30.2 a	60.2 b	9.6 a	36.2 b
All	All	2017	All	Industrial	12.6 b	19.9 b	69.7 a	10.4 a	28.6 a
Anvergur	IND	All	All	All	11.4 b	28.1 a	65.6 ab	6.3 b	-
Bidi17	FAR	All	All	All	13.7 a	29.0 a	62.6 bc	8.4 ab	-
Claudio	IND	All	All	All	12.1 b	26.8 a	61.3 c	11.8 a	-
LA1823	FAR	All	All	All	12.2 b	26.2 a	68.3 a	5.5 b	-
All	FAR	All	All	All	12.9 a	27.6 a	65.5 a	6.9 a	-
All	IND	All	All	All	11.7 b	27.5 a	63.5 a	9.1 a	-
All	All	All	All	Artisanal_1_2	11.9	33.3	60.3	6.4	-

*Values followed by the same letter within each column are not significantly different based on Student-Newman-Keuls (SNK) test performed at α = 0.05); (-) data not available
